# Easy mammalian expression and crystallography of maltose-binding protein-fused human proteins

**DOI:** 10.1016/j.jsb.2016.01.016

**Published:** 2016-04

**Authors:** Marcel Bokhove, Hamed Sadat Al Hosseini, Takako Saito, Elisa Dioguardi, Katharina Gegenschatz-Schmid, Kaoru Nishimura, Isha Raj, Daniele de Sanctis, Ling Han, Luca Jovine

**Affiliations:** a Karolinska Institutet, Department of Biosciences and Nutrition & Center for Innovative Medicine, Huddinge, Sweden; b ESRF – The European Synchrotron, Grenoble 38000, France

**Keywords:** AKH, adipokinetic hormone, CHO, Chinese hamster ovary, Crypα, cell adhesion molecule-like tyrosine phosphatase α, Endo H, Endoglycosidase H, ENG, endoglin/CD105, HEK, human embryonic kidney, IMAC, immobilized metal affinity chromatography, MBP, maltose-binding protein, mMBP, mammalianized maltose-binding protein, ORF, open reading frame, PDB, Protein Data Bank, PEI, polyethylenimine, PNGase F, peptide *N*-glycosidase F, SEC, size-exclusion chromatography, TEV, tobacco etch virus, TEV_CS_, tobacco etch virus protease cleavage site, UMOD, uromodulin, VERL, vitelline envelope receptor for lysin, Mammalian cell expression, Glycoproteins, Maltose-binding protein fusion, Crystallization, X-ray crystallography, Molecular replacement

## Abstract

We present a strategy to obtain milligrams of highly post-translationally modified eukaryotic proteins, transiently expressed in mammalian cells as rigid or cleavable fusions with a mammalianized version of bacterial maltose-binding protein (mMBP). This variant was engineered to combine mutations that enhance MBP solubility and affinity purification, as well as provide crystal-packing interactions for increased crystallizability. Using this cell type-independent approach, we could increase the expression of secreted and intracellular human proteins up to 200-fold. By molecular replacement with MBP, we readily determined five novel high-resolution structures of rigid fusions of targets that otherwise defied crystallization.

## 1 Introduction

X-ray crystallography is 100 years old and great advances have been made in structural biology since the first protein structures appeared in the fifties. However, because the folding and function of many biologically and medically relevant human proteins is dependent on their correct post-translational modification, these molecules remain difficult to produce in bacteria and thus hard to investigate biochemically and structurally. Most strikingly, although more than 50% of native proteins are glycosylated and this is often crucial for their activity, less than 5% of Protein Data Bank (PDB) structures contain covalently-bound carbohydrate chains (Lütteke and von der Lieth, 2009). In the case of human proteins, which account for more than 30% of the PDB, under-representation of post-translationally modified structures is largely a consequence of the fact that approximately 70% of unique PDB chains derive from bacterially-expressed material (Berman et al., 2000).

Mammalian cells are in principle the expression system of choice for producing properly folded, biologically active human proteins. However, this approach is still seldom used in structural biology because of the time required for stable cell line generation, the high price of commercial formulations for transient transfection and the limited yield of recombinant protein. Adoption of polyethylenimine (PEI) as a transfection reagent has considerably reduced costs and time consumption (Aricescu et al., 2006), and fusion to highly expressed mammalian carriers secreted by stable lines (Leahy et al., 2000, Lo et al., 1998) as well as lentiviral transduction (Bandaranayake et al., 2011) can increase protein yields. Nevertheless, proteolytic separation of mammalian fusion partners is required for structural studies, and general use of lentivirus-based systems is hindered by biosafety and construct size limitations. T4 lysozyme fusions have also been exploited successfully, but their use has so far only been applied to G protein-coupled receptors (Thorsen et al., 2014). Moreover, in the absence of previous structural information, crystallographic phasing of proteins expressed in mammalian cells still largely relies on the use of heavy atom soaks, anomalous signal derived from sulfur-containing Cys and Met residues or – albeit more rarely due to cell toxicity and reduced yield – selenomethionine incorporation.

Using a horizontal synthetic gene transfer approach, we investigated whether fusion to MBP – a highly successful bacterial expression method (di Guan et al., 1988, Lebendiker and Danieli, 2011) – could be applied in mammalian cells in order to solve the aforementioned issues. MBP is a powerful solubility enhancer that often helps folding of fusion partners (Needle and Waugh, 2014), can be used both as a detection and a purification tag (di Guan et al., 1988, Lebendiker and Danieli, 2011), and has been exploited to facilitate the crystallization and phasing of an increasing number of rigidly fused passenger proteins (Center et al., 1998, Cherry et al., 2013, Kobe et al., 2015, Laganowsky et al., 2011, Monné et al., 2008, Moon et al., 2010, Smyth et al., 2003, Waugh, 2015).

In this manuscript we report that fusion to MBP significantly enhances mammalian cell expression of complex eukaryotic proteins. This strategy not only increases the solubility and crystallizability of passenger proteins, but also provides powerful phasing information for rapidly determining target structures by molecular replacement.

## 2 Methods

### 2.1 DNA constructs

A DNA fragment encoding mMBP and a multiple cloning site was synthesized (GenScript) and subcloned into pHLsec (Aricescu et al., 2006), resulting in pHLmMBP construct 1 (Fig. S1A). All subsequent constructs were generated by PCR cloning using PfuTurbo DNA polymerase (Agilent Technologies). Restriction site information for cloning inserts in frame with sequences encoding the cell adhesion molecule-like tyrosine phosphatase α (Crypα) signal peptide, mMBP, tobacco etch virus (TEV) protease cleavage site and/or affinity purification/detection tags may be found in Fig. S1A and B.

Non-mMBP fused constructs were prepared by cloning cDNAs in frame with the Crypα-encoding sequence of pHLsec using AgeI and XhoI restriction enzymes. The cDNA encoding ENG-N with its native signal peptide sequence was cloned between the EcoRI and EcoRV restriction sites of pHLsec. Unfused N-glycosylation site mutants of the polymerization region of UMOD were expressed with an immunoglobulin-kappa signal peptide using pIRES2-EGFP (Clontech).

For insect cell expression, 6His-mMBP-UMODp_XR_ DNA was cloned in frame with the adipokinetic hormone (AKH) signal peptide-encoding sequence of pIEX-5 (Novagen/Merck Millipore), whereas Crypα-6His-mMBP-UMODp_XR_ DNA was cloned into pIEX-4 (Novagen/Merck Millipore).

### 2.2 Protein expression

DNA for transfection was prepared using HiSpeed Plasmid Midi/Maxi kits or EndoFree Plasmid Giga kits (QIAGEN). Human embryonic kidney (HEK) 293T or 293S (ATCC CRL-3022) cells were amplified, transferred to a suitable expression vessel and transiently transfected using 25 kDa branched PEI, based on a published protocol (Aricescu et al., 2006). Fresh DMEM medium supplemented with 4 mM L-Gln (Thermo Fisher Scientific) and – if required – 2% fetal bovine serum (Biological Industries) was added to 90–95% confluent cells using the volumes specified in Table S1 for transfection of 6-well plates (10 cm^2^/well; Corning), T-flasks (150 cm^2^; BD Biosciences), ribbed roller bottles (2125 cm^2^; Greiner) or cell factories (2528 cm^2^; Thermo Fisher Scientific). Transfection mixes were prepared by first combining serum-free medium and DNA, then adding PEI, and finally incubating the resulting solution for 10 min at 20 °C before adding it to the cells. Unlike HEK293T cells, HEK293S cells tend to detach from ribbed roller bottles and were thus preferentially grown in T-flasks or cell factories. Medium was harvested three days after transfection.

For intracellular protein production, 200 ml FreeStyle 293-F HEK cells (HEK293F; Thermo Fisher Scientific) were grown in suspension in FreeStyle 293 Expression Medium (Thermo Fisher Scientific) in a 1.0 L plastic shake flask (Corning), using a shaking 8% CO_2_ incubator. When cells reached a density of 2.5–3.0 × 10^6^ cells/ml, they were transiently transfected by diluting them 1:1 with medium containing 3.0 μg/ml plasmid DNA and 9.0 μg/ml 25 kDa branched PEI. Harvesting was performed four days after transfection.

Transient transfections of Chinese hamster ovary (CHO)-K1 cells (American Type Culture Collection) were performed in 6-well plates with Lipofectamine 2000 (Thermo Fisher Scientific), essentially according to the manufacturer’s instructions.

Sf9 insect cells (Novagen/Merck Millipore) were amplified and transferred to 6-well plates in Sf-900 II SFM medium (Thermo Fisher Scientific) supplemented with 5% fetal bovine serum. Transient transfections were performed using 40 kDa linear PEI (Polysciences) as transfection reagent. 2 μg DNA was combined with 100 μl Sf-900 II SFM medium and the same was done for 10 μg PEI. DNA/PEI complex formation was performed by combining both mixes, followed by incubation for 15 min at room temperature. After replacing the cell medium with Sf-900 II SFM, the transfection mix was added drop-wise to the cells. Harvesting was performed three days after transfection.

### 2.3 Protein detection

Immunoblotting was performed with Penta·His mouse monoclonal antibody (1:1000; QIAGEN) and horseradish peroxidase-conjugated goat anti-mouse IgG (1:10,000; Jackson ImmunoResearch Laboratories). Chemiluminescence detection was performed with Western Lightning ECL Plus (Perkin Elmer). Densitometric analysis was carried out with ImageJ (Schneider et al., 2012).

### 2.4 Protein purification and deglycosylation

Conditioned medium containing secreted mMBP fusions was adjusted to 10 mM imidazole, 150 mM NaCl and 20 mM Na-HEPES pH 8.0 (immobilized metal affinity chromatography (IMAC) binding buffer I). 10 ml pre-equilibrated Ni–NTA agarose slurry (QIAGEN) was then added per liter of medium and allowed to incubate overnight at 4 °C using an overhead shaker (Heidolph). Beads were collected and washed with 20 column volumes of IMAC binding buffer I. Proteins were batch-eluted with 5 column volumes of 20 mM Na-HEPES pH 8.0, 150 mM NaCl, 500 mM imidazole, and concentrated using centrifugal filtration devices (Amicon). In the case of glycoproteins expressed in HEK293S cells, concentrated mMBP fusions were deglycosylated with Endoglycosidase H (Endo H; 1:10 mass ratio) for 1 h at 37 °C in 120 mM Na/K phosphate pH 6.0 (Sigma–Aldrich). Concentrated material was applied to a Superdex 200 26/600 size-exclusion chromatography (SEC) column attached to an ÄKTAFPLC chromatography system (GE Healthcare) and pre-equilibrated with 100 mM NaCl, 20 mM Na-HEPES pH 8.0, 10 mM D-maltose (Sigma–Aldrich). Fractions containing mMBP fusion proteins were pooled, concentrated and used for crystallization trials. For purification from smaller cell culture vessels, the same protocol was scaled down linearly.

To improve sample purity and assess relative binding affinity, a more stringent IMAC bead wash step with 20 mM imidazole was performed during purification of fusions with extended N-terminal His-tags (pHLmMBP vectors 3–5; Figs. S1A and S3).

Affinity purification of intracellularly-produced human GLI fusion was enhanced by exploiting the amylose-binding properties of mMBP. HEK293F cells were harvested and lysed at 4 °C using 20 mM Na-HEPES pH 8.0, 50 mM NaCl, 1 mM MgCl_2_ (IMAC binding buffer II) supplemented with 1% Triton X-100 (Sigma–Aldrich) and EDTA-free protease-inhibitor cocktail (Roche). Cell debris was removed by centrifugation at 4000 x *g* for 20 min, followed by filtration using a 0.22 μm filter. Cleared cell lysate was incubated with 12.5 ml amylose beads (New England Biolabs) per liter at 4 °C overnight to bind fusion protein. After washing with 5 column volumes of IMAC binding buffer II, the mMBP fusion was batch-eluted with 5 column volumes of IMAC binding buffer II supplemented with 20 mM D-maltose (Sigma–Aldrich). Amylose affinity chromatography was followed by IMAC and optionally SEC as described above.

### 2.5 mMBP removal

To separate passenger proteins from mMBP, an 18-residue linker (GGGSGGGSENLYFQSAAA) was engineered between the two fusion moieties that is resistant to aspecific proteolysis by cellular proteases, but can be efficiently cleaved by TEV protease (pHLmMBP vectors 8–11; Fig. S1A).

For TEV protease digestion experiments, mMBP-fused UMODp_XR_ was expressed using vector 10 (Fig. S1A). IMAC-eluted protein was concentrated to approximately 1 mg/ml and the buffer was adjusted to 10 mM Na-HEPES pH 8.0, 50 mM NaCl. Fusion protein was digested overnight at 20 °C with TEV protease (Tropea et al., 2009), using a mass ratio of 1:100 (TEV protease:mMBP fusion). Digested material was then deglycosylated overnight at 37 °C, using 4–8 units peptide *N*-glycosidase F (PNGase F; New England Biolabs) per μg fusion protein. For cleavage of mMBP-GLI produced intracellularly using vector 8 (Fig. S1A), a 1:10 mass ratio (TEV protease:mMBP fusion) was used. Digestion products were analyzed by SDS–PAGE (Fig. 4) and band identity was verified by immunoblot (Fig. S5).

### 2.6 Crystallization

**Fig. 1:**
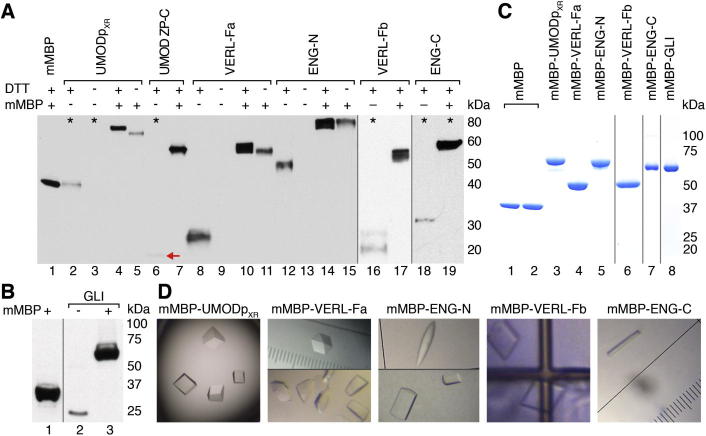
Mammalian expression, purification and crystallization of mMBP-fused target proteins. (A) Immunoblot with monoclonal anti-5His of unfused and mMBP-fused proteins secreted from HEK293T cells. All constructs carry a 3Ala linker after mMBP and a 6His-tag at either terminus. Formation of intramolecular disulfides is indicated by a mobility shift between reducing and non-reducing conditions. 5 μl of medium were loaded, except in lanes marked with an asterisk (20 μl). A red arrow marks the position of UMOD ZP-C domain, which is barely detectable when expressed in unfused form. (B) Reducing anti-5His immunoblot of proteins expressed in the cytoplasm of HEK293F cells. (C) Non-reducing Coomassie-stained gel of 3 μg purified mMBP produced by either HEK293T (lane 1) or HEK293S cells (lane 2), as well as purified secreted (lanes 3–7) or intracellular (lane 8) mMBP fusion proteins. HEK293S-derived fusions (lanes 3, 5 and 6) were treated with Endo H during purification. (D) Crystals of mMBP-fused proteins.

Sitting-drop vapor diffusion crystallization trials were performed with a mosquito crystallization robot (TTP Labtech) using NeXtal DWBlock (QIAGEN) and Morpheus HT-96 (Molecular Dimensions) (Gorrec, 2009) crystallization screens. Crystallization trials were performed both in the presence and absence of Zn(OAc)_2_ in a 1:1.5 molar ratio. Purified mMBP-UMODp_XR_ was concentrated to 6.5–15.0 mg/ml and crystallized with Zn(OAc)_2_ in 900 mM Na/K tartrate, Tris–HCl pH 7.0–8.6 at 20 °C. Crystals grew to approximately 300 μm (Fig. 1D) and were then adjusted to 4 °C, after which they were transferred stepwise to mother liquor supplied with 20–30% glycerol before flash cooling in liquid nitrogen. Crystals of mMBP-fused VERL-Fa, ENG-N and VERL-Fb (Fig. 1D) readily appeared in both polyethylene glycol- and salt-based conditions; crystals of mMBP-ENG-C (Fig. 1D) grew using a PEG/MPD precipitant mixture. Notably, crystals of mMBP-VERL-Fa could be cryoprotected for data collection using a stabilization solution supplemented with 30% maltose, suggesting that the latter may also work as a cryoprotectant for other MBP fusions.

### 2.7 X-ray diffraction data collection and structure determination

Data were collected at 100 K at beamlines ID29 (de Sanctis et al., 2012) and ID23-1 (Nurizzo et al., 2006) of the European Synchrotron Radiation Facility (ESRF, Grenoble), equipped with DECTRIS PILATUS 6M-F detectors. 0.1-degree oscillation frames were integrated and scaled with XDS (Kabsch, 2010).

**Fig. 2:**
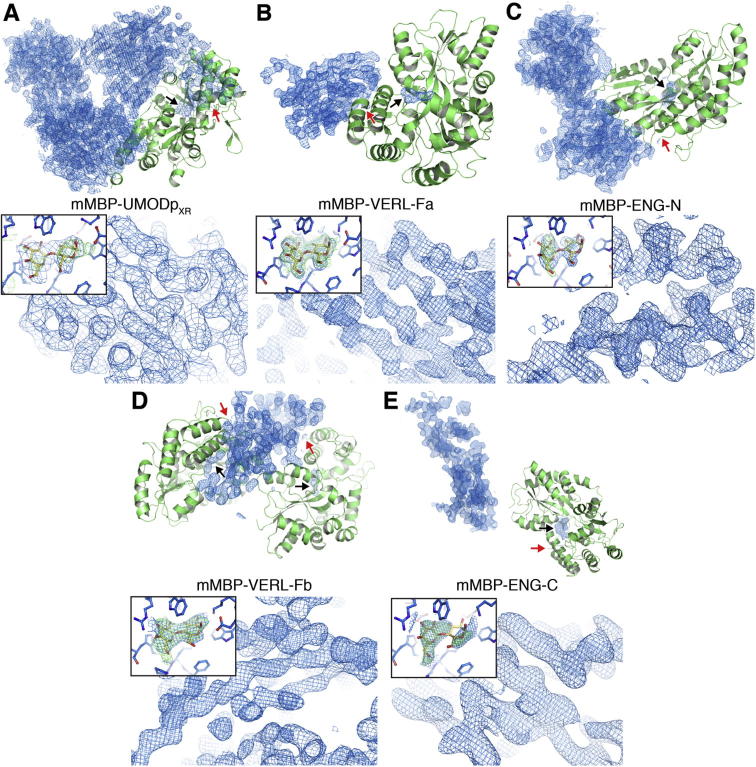
Target protein phasing by molecular replacement with mMBP. Top halves of the panels show mMBP molecular replacement solutions in cartoon representation. σA-weighted, density-modified electron density maps of passenger proteins obtained after molecular replacement are colored blue and contoured at 1.0 σ. Red arrows point to the C-terminus of mMBP to which each passenger protein is fused; black arrows indicate maltose electron density. Bottom halves of the panels are close-ups of the electron density maps in the top halves, with readily interpretable secondary structure features. Insets show molecular replacement electron density maps (1.0 σ, blue) and difference maps (2.5 σ, green) of the mMBP maltose-binding pocket.

**Fig. 3:**
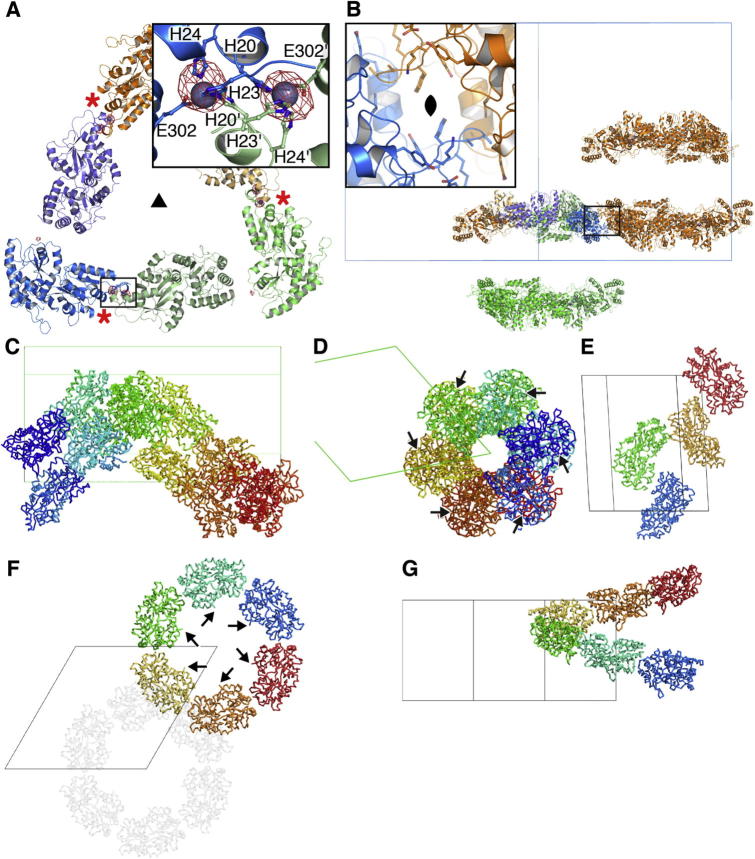
mMBP-induced packing in UMODp_XR_, VERL-Fa, VERL-Fb and ENG-C fusion crystals. (A) The N-terminal His-tags of two molecules, together with a Glu residue of mMBP and two Zn^2+^ ions, mediate crystal-packing interactions of mMBP-UMODp_XR_. Crystals could only be obtained in the presence of zinc, and identity of the ion was confirmed by an anomalous difference map (5.0 σ, red) generated from data collected at the zinc absorption edge (*λ* = 1.28215 Å). Red asterisks show the location of the crystal contact, whose details are shown in the inset, and the triangle indicates the resulting 3-fold crystallographic axis in the *H*32 space group. Only the mMBP moieties of fusion proteins are shown here and in subsequent panels. (B) A crystallographic 2-fold generates a hexamer from the trimer in A, shown here in side view. Two such hexamers pack through two surface loops that contain entropy reduction mutations (Ala196-Ala197, equivalent to Ala172-Ala173 in Fig. S1). The inset shows an enlarged view of the loop-loop interaction, which forms the major interaction throughout the crystal. (C and D) Orthogonal views of mMBP packing in crystals of mMBP-fused VERL-Fa. mMBP runs as a spiral through the crystal, which has space group *P*6_5_22. (E) mMBP also provides packing interactions to mMBP-fused dimers of VERL-Fb in space group *P*2_1_. (F and G) Orthogonal views of mMBP-induced packing interactions in crystals of mMBP-fused ENG-C. mMBP forms stacked spirals that run through the crystal with space group *P*6_5_. The arrows in (D) and (F) indicate the C-terminus of mMBP where the target protein sequence starts, protruding outwards.

Molecular replacement was performed with Phaser (McCoy et al., 2007), using an ensemble of MBP PDB entries 3SET and 3SEX (Laganowsky et al., 2011) as the search model. After obtaining initial molecular replacement phases, density modification was performed with RESOLVE (Terwilliger, 2004) in the PHENIX package (Adams et al., 2010) (Fig. 2). Model building was carried out using Buccaneer (Cowtan, 2006), phenix.autobuild (Terwilliger et al., 2008) and Coot (Emsley et al., 2010); refinement and validation were performed with phenix.refine (Afonine et al., 2012) and MolProbity (Chen et al., 2010), respectively. Fig. 2, Fig. 3 were created with PyMOL (Schroedinger, LLC).

## 3 Results

### 3.1 Design of an MBP fusion expression system for mammalian cells

The primary structure of MBP suggests that this protein could be an optimal fusion partner for expression in mammalian cells, particularly in the case of secreted targets. This is because, despite its considerable mass (∼40 kDa), MBP lacks sites that could be spuriously N-glycosylated, as well as cysteine residues that may interfere with the correct formation of disulfide bonds in the passenger. Furthermore, MBP is a globular protein rich in α-helices, making it a powerful search model for molecular replacement (Smyth et al., 2003, Waugh, 2015).

Numerous amino acid changes reported to increase MBP solubility, maltose binding affinity and crystallizability were combined into a synthetic open reading frame (ORF) codon-optimized for mammalian cells (mMBP; Fig. S1B). These include A312V and I317V, a mutation pair that was found to positively impact expression and purification of MBP fusion proteins in *Escherichia coli* by significantly increasing the yield of MBP and its affinity for maltose (Walker et al., 2010). Mutations E359A, K362A and D363A were also introduced, which can facilitate fusion crystallization by reducing the flexibility of the linker between MBP and passenger proteins (Center et al., 1998), as well as allowing the two moieties to pack against each other (Monné et al., 2008). Other changes were based on surface entropy reduction, an approach that enhances protein crystallizability by decreasing surface flexibility (Cooper et al., 2007). This is achieved by mutation of exposed charged residues to alanine, and application of this strategy to MBP showed that mutations D82A, K83A, E172A, N173A and K239A significantly enhanced crystallization of several MBP fusions (Moon et al., 2010). Finally, mutation to histidine of α-helical residue pairs located four positions apart, such as A215/K219, K25/K29 and E309/K313, was recently used to engineer half-binding sites for divalent metal ions in MBP. Remarkably, the resulting synthetically symmetrized variants were found to highly enhance crystallization by providing metal-dependent crystal-packing interactions between neighboring MBP molecules (Laganowsky et al., 2011). Because of its compatibility with a wide variety of crystal contacts (Laganowsky et al., 2011) and based on previous successful usage in our laboratory (Cherry et al., 2013), we decided to introduce mutation pair A215H/K219H in our synthetic construct.

Different configurations of signal peptide- and/or tag-encoding DNA sequences were assembled in frame with the basic mMBP ORF and cloned into transient expression vector pHLsec (Aricescu et al., 2006). This generated a family of plasmids expressing mMBP fusion constructs that can either be secreted or produced intracellularly and contain affinity and detection tags at either terminus (pHLmMBP; Fig. S1A).

To test whether mammalian cells could efficiently produce mMBP, we used PEI to transiently transfect HEK293 cells with a construct driving secretion of unfused, His-tagged mMBP. The protein could be detected in unconcentrated conditioned medium (Fig. 1A, lane 1) and purified to homogeneity by batch IMAC and SEC, with a final yield of 15 mg/l (HEK293T cells) or 6 mg/l (HEK293S cells) (Fig. 1C, lanes 1 and 2). By lacking *N*-acetylglucosaminyltransferase I, the HEK293S cell line is incapable of synthesizing complex N-glycans (Reeves et al., 2002). As a result, it secretes Man_5_GlcNAc_2_-glycosylated proteins that can be processed with Endo H to obtain homogeneous material suitable for crystallographic studies (Chang et al., 2007).

### 3.2 Production of mMBP-fused proteins

To evaluate if mMBP could help mammalian production of passenger proteins, secreted fusion constructs were generated for several difficult extracellular targets that did not yield material suitable for structural studies due to low expression, reduced solubility or lack of diffracting crystals. These included UMODp_XR_, a soluble variant of the polymerization region of human uromodulin (UMOD)/Tamm–Horsfall protein, a urinary molecule involved in kidney protection and hypertension (Rampoldi et al., 2011, Trudu et al., 2013); the zona pellucida (ZP)-C domain of UMOD; a fragment of vitelline envelope receptor for lysin (VERL-Fa) (Swanson and Vacquier, 1997); and the N- or C-terminal region of human endoglin/CD105 (ENG-N and ENG-C), a type I transmembrane glycoprotein involved in angiogenesis (Gougos and Letarte, 1990). C-terminal fusion to mMBP via a 3-alanine linker significantly boosted the expression of all targets, up to 200-fold in the case of UMOD ZP-C (Fig. 1A). Comparable results were obtained for VERL-Fb (another fragment of VERL that shares 40% sequence identity with VERL-Fa) and ENG-C (Fig. 1A), as well as by fusing intracellular proteins (such as cancer-associated human transcription factor GLI (Kinzler et al., 1988)) to a non-secreted version of mMBP (Fig. 1B).

Whereas 3.1 mg of an N-glycosylation mutant of mMBP-VERL-Fa and 2.6 mg of mMBP-ENG-C could be purified from 1 l of transiently transfected HEK293T cell medium (Fig. 1C, lanes 4 and 7), mMBP-UMODp_XR_, ENG-N and VERL-Fb were expressed in fully glycosylated form. N-glycosylation is essential for secretion of both the polymerization region of UMOD (Fig. S2) and ENG-N (Gregory, 2011). After Endo H treatment of HEK293S-derived material, we obtained 3 mg/l, 7 mg/l and 9 mg/l of pure mMBP-UMODp_XR_, mMBP-ENG-N and mMBP-VERL-Fb, respectively (Fig. 1C, lanes 3, 5 and 6). Intracellular expression of mMBP-GLI using HEK293F cells yielded 2.5 mg/l of purified material (Fig. 1C, lane 8).

### 3.3 Crystallization and structure determination of mMBP-fused proteins

mMBP fusions of UMODp_XR_ as well as of VERL-Fa, ENG-N, VERL-Fb and ENG-C readily produced multiple diffraction-quality crystal forms when screened at 6–20 mg/ml concentrations (Fig. 1D). A native dataset collected from a single crystal of mMBP-UMODp_XR_ was processed to 3.2 Å resolution, with overall *R*_pim_ and CC_1/2_ values of 3.8% and 99.9%, respectively (outer shell CC_1/2_: 43.2%). Crystals of mMBP fusions of VERL-Fa, ENG-N, VERL-Fb and ENG-C diffracted to resolutions of 1.8 Å, 2.4 Å, 2.9 Å and 2.7 Å, with *R*_pim_ and CC_1/2_ values of 2.1% and 100.0% (62.3%), 4.6% and 99.9% (52.0%), 9.9% and 99.0% (50.3%), 6.2% and 99.8% (66.2%), respectively.

Molecular replacement using MBP as a search model yielded translation function Z-scores of 27.7 (mMBP-UMODp_XR_), 58.2 (mMBP-VERL-Fa), 22.6 (mMBP-ENG-N), 39.9 (mMBP-VERL-Fb) and 30.1 (mMBP-ENG-C). Statistical density modification produced maps that showed clear secondary structure for the passenger moiety of the fusions; moreover, the mMBP binding pocket contained difference density for maltose, which was added during purification but coordinates for which were not included in the search model (Fig. 2).

All structures could be almost entirely autobuilt, and mMBP-UMODp_XR_, whose mMBP phasing model corresponded to only 28% of the ∼150 kDa asymmetric unit content, has been refined to *R* = 22.1%, *R*_free_ = 24.6% (PDB ID 4WRN; Bokhove et al., 2016). Refinement of the other structures is ongoing, and biological implications will be described elsewhere. Notably, most of the crystal packing interactions observed in the five structures are mediated by mMBP; in particular, some involve loop residues mutated to lower the surface entropy and increase the crystallizability of the protein, while others depend on contacts made by mMBP, its N-terminal His-tag and zinc ions from the mother liquor (Fig. 3).

### 3.4 Cleavable mMBP fusions and expression in other cell lines

**Fig. 4:**
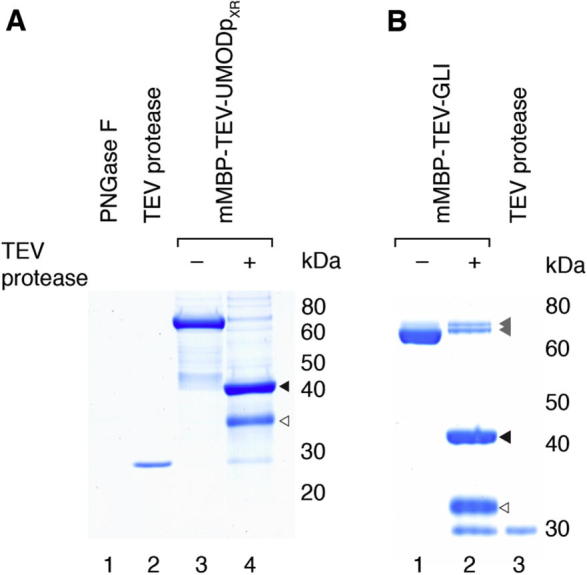
Cleavage of secreted and intracellular mMBP fusions with a TEV protease-sensitive linker. (A) Coomassie SDS–PAGE analysis of a TEV protease digestion experiment to release UMODp_XR_ from the mMBP fusion partner. Protein was partially purified by IMAC, digested with TEV protease and deglycosylated with PNGase F; to compensate for the loss of mass, the amount of cleaved material loaded was double that of the fusion. White and black arrowheads indicate deglycosylated UMODp_XR_ and mMBP, respectively. Lanes 1 and 2 are control PNGase F and TEV protease. (B) Coomassie SDS–PAGE analysis of TEV protease cleavage of intracellularly expressed mMBP-GLI fusion. Material was affinity-purified using amylose resin followed by IMAC. White arrowhead: GLI; black arrowhead: mMBP; gray arrowheads: uncleaved mMBP-GLI. Lane 3: control TEV protease.

For functional studies as well as crystallographic analysis of targets that have structural homologues in the PDB, maintaining proteins of interest as fusions with mMBP may not always be optimal. For such cases, we constructed expression vector variants that encode cleavable linkers (pHLmMBP vectors 7–11; Fig. S1A), which allow efficient removal of the tag from both secreted and intracellular fusions (Fig. 4).

Yield and purity of cleavable fusions can be further improved by increasing the length of the N-terminal His-tag to 8 residues (Fig. S3) and/or by adding a spacer between mMBP and the cleavage site to facilitate an additional mMBP-mediated amylose affinity purification step (Fig. 4B). Importantly, the mMBP fusion system can also be used in CHO-K1 cells, where fusion of UMODp_XR_ to mMBP increased expression by a factor of six compared to unfused UMODp_XR_ (Fig. S4A). Finally, transiently transfected Sf9 insect cells also efficiently produce and secrete mMBP-fused proteins (Fig. S4B), demonstrating the remarkable versatility of this bacterially-derived fusion system. Notably, the mammalian Crypα signal peptide is as efficient as its insect AKH-derived counterpart to drive mMBP fusion protein secretion by Sf9 cells (compare lanes 3, 5 with 2, 4). This suggests that – at least for initial screening – mMBP fusion construct ORFs developed for mammalian cell expression can be directly subcloned into insect expression vectors with their Crypα-encoding region.

## 4 Discussion

The high yield of mMBP fusion proteins not only largely meets the demands of functional and biochemical studies, but also constitutes a significant advantage for the structural investigation of challenging targets expressed in mammalian cells. By combining the post-translational modification machinery of HEK293 cells with the ability of mMBP to both facilitate crystal packing and phase “real-life” X-ray diffraction data, we were able to determine the structure of five complex eukaryotic proteins. Especially the crystal structure of mMBP-UMODp_XR_ revealed a striking mMBP-induced crystal-packing interface in which the N-terminal His-tag and a glutamate residue from mMBP coordinate zinc ions from the crystallization solution (Fig. 3A). This observation suggests that crystallization trials of mMBP fusion proteins should also routinely be performed in the presence of Zn^2+^, which may additionally be exploited for experimental phasing (Cha et al., 2012).

Notably, the use of mMBP fusions was essential for all these projects because none of the protein targets folded correctly in *E. coli* nor could they be crystallized when expressed in isolated form in mammalian cells. In the future, mammalian protein production exploiting the mMBP fusion system could be further improved by transfecting new highly expressing cell types, such as PER.C6 and HKB-11 (Swiech et al., 2012). Finally, our strategy could in principle be combined with other powerful emerging technologies such as Daedalus, a lentiviral vector-based expression system (Bandaranayake et al., 2011) and baculovirus-mediated gene transduction of mammalian cells (BacMam) (Dukkipati et al., 2008).

## 5 Plasmids

mMBP fusion mammalian expression vectors pHLmMBP-1, 2, 3, 6, 8, 10 and 11 (Fig. S1A) have been made available to the scientific community through Addgene (http://www.addgene.org; accession numbers 72343-72349).

## Appendix A Supplementary data

Supplementary data This file contains supplementary figures and tables.
