# Supplementary material for: Easy mammalian expression and crystallography of maltose-binding protein-fused human proteins
Source: J Struct Biol. 2016 Apr;194(1):1–7. doi: 10.1016/j.jsb.2016.01.016 (PMC4771870; doi:10.1016/j.jsb.2016.01.016)
Supplement: Supplementary data — This file contains supplementary figures and tables. [file mmc1.pdf]

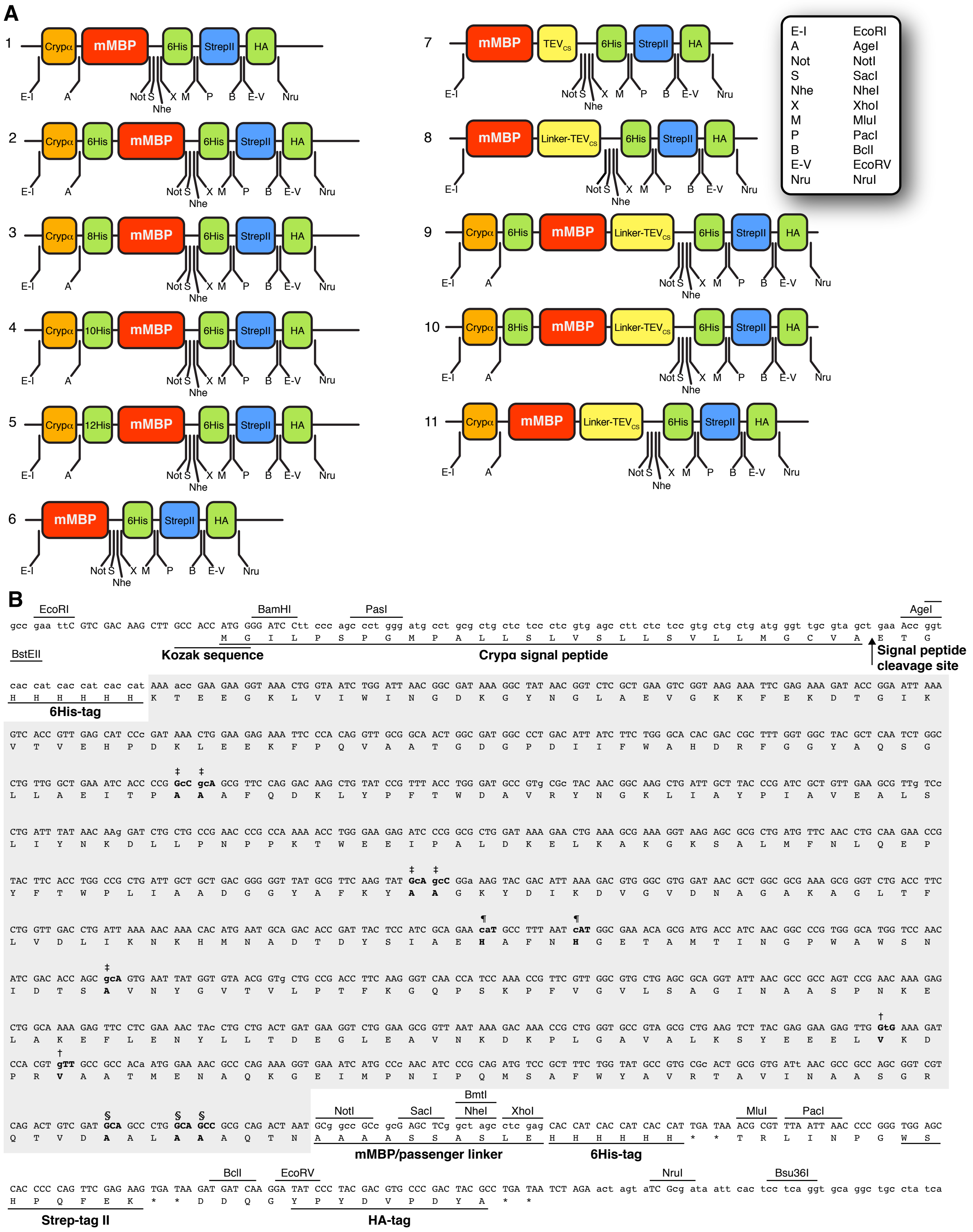

Fig. S1. mMBP fusion configurations and pHLmMBP expression vector features. (A) Schematic representation of constructs used for secreted or intracellular expression. Elements are represented by boxes and restriction site positions are indicated. Crypα: chicken cell adhesion molecule-like tyrosine phosphatase CRYP α signal peptide with an R2G mutation (Aricescu et al., 2006); TEV_{CS}: TEV protease cleavage site. (B) mMBP coding region details of representative expression vector 2, including optimization mutations (†: A312V, I317V (Walker et al., 2010); §: E359A, K362A, D363A (Center et al., 1998); ‡: D82A, K83A, E172A, N173A, K239A (Moon et al., 2010); ¶: A215H, K219H (Laganowsky et al., 2011)).

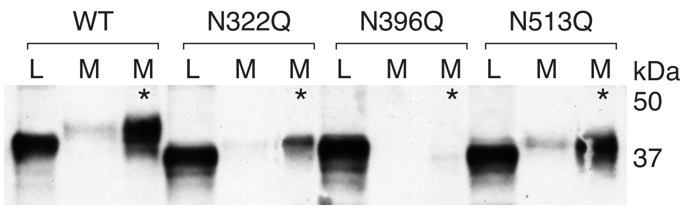

Fig. S2. N-glycosylation site mutation severely impairs secretion of the polymerization region of UMOD. Anti-5His immunoblot of cell lysates (L) and conditioned media (M) indicates that, although all constructs are produced, protein secretion is impaired when N-glycosylation of UMOD N322 or N396 is abolished. 20 µl of medium were loaded, except in lanes marked with an asterisk (100 µl).

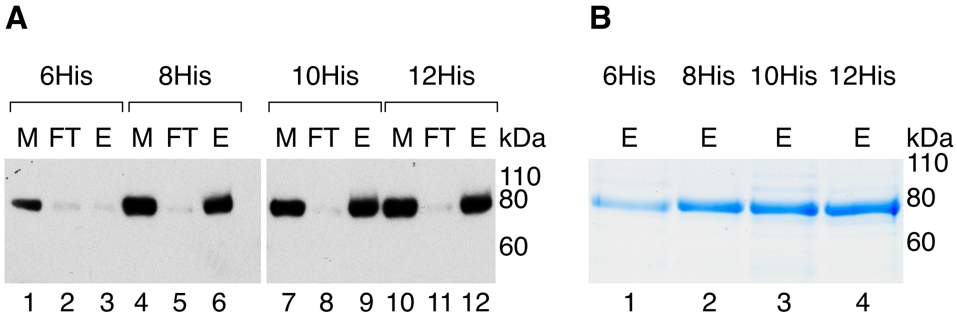

Fig. S3. Expression of mMBP-fused UMODp$_{XR}$ constructs with extended N-terminal His-tags. (A) Immunoblot analysis with monoclonal anti-5His of a pull-down experiment using IMAC beads. Amounts of material equivalent to 15 µl of input conditioned medium (M) were loaded for both flow-through (FT) and elution (E) fractions. Using 20 mM imidazole in the binding buffer to prevent aspecific interactions with the IMAC beads, 6His-tagged material binds poorly to the resin (lane 3). However, extension of the N-terminal His-tag significantly increases pull-down efficiency in the same conditions (lanes 6, 9 and 12). (B) Coomassie SDS-PAGE analysis of IMAC elution fractions comparable to those shown in A and equivalent to 500 µl of input medium.

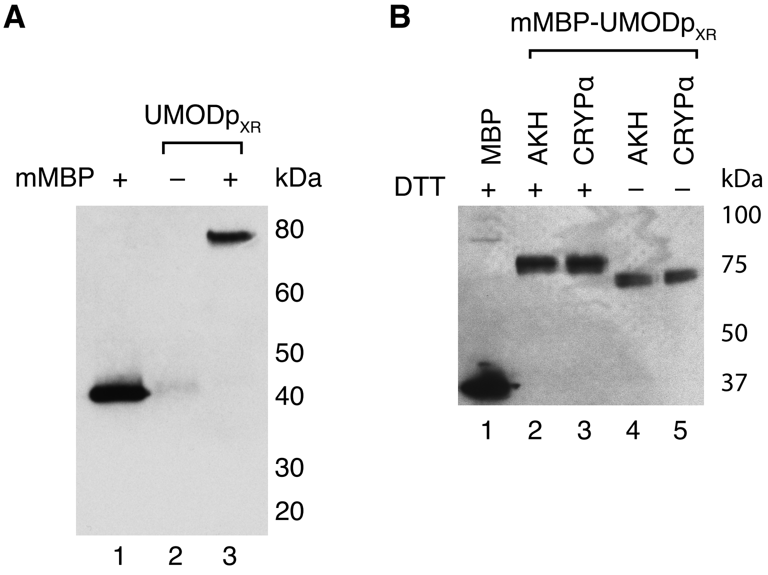

Fig. S4. Application of the mMBP fusion system to other eukaryotic expression systems. (A) Anti-5His immunoblot shows that mMBP is efficiently secreted into the medium of CHO-K1, a mammalian cell line used extensively for protein production (lane 1). As in HEK293 cells, secreted expression of UMODp$_{XR}$ is highly increased upon fusion to mMBP (compare lanes 2 and 3). (B) Anti-MBP immunoblot shows Sf9 insect cell secretion of mMBP-fused UMODp$_{XR}$ driven by either insect AKH (lanes 2 and 4) or mammalian Crypα (lane 3 and 5) signal peptides. The mobility shift between reducing and non-reducing lanes indicates that intramolecular disulfide bonds are formed. Densitometric comparison with lane 1 (500 ng *E. coli*-produced MBP) indicates that 6 µg and 8 µg mMBP-UMODp$_{XR}$ are secreted per ml of Sf9 medium using the AKH and Crypα signal peptides, respectively.

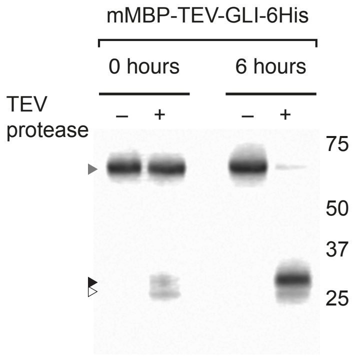

Fig. S5. Identification of TEV protease digestion products of mMBP-TEV-GLI. Anti-5His immunoblot analysis of cleavage experiments of intracellularly expressed mMBP-TEV-GLI fusion with TEV protease, at the onset (0 hours) and after 6 hours digestion. White arrowhead: TEV; black arrowhead: GLI; grey arrowheads uncleaved mMBP-TEV-GLI.

Table S1. Composition table to perform transient transfections in multiple cell culture vessels

	Fresh medium (ml)	Transfection mix		
		Serum-free medium + DNA + PEI (ml)	DNA (µg)	PEI (µg)
**6-well**	0.94	0.240	2.35	5.0
**T-flask**	18.7	3.67	36.0	72.0
**Roller bottle**	200.0	51.0	500.0	1000.0
**Cell factory**	315.0	61.2	600.0	1200.0

Transfection mix composition should be chosen such that the final volume matches the final volume of serum-free medium + DNA + PEI. During PEI:DNA complex-formation spent medium can be replaced with fresh medium.
